# Emodin inhibits zinc-induced neurotoxicity in neuroblastoma SH-SY5Y cells

**DOI:** 10.1042/BSR20182378

**Published:** 2019-05-14

**Authors:** Wenzhou Liu, Zhen Fan, Feng Gao, Li Ou, Min Li, Xin Zhou, Wenjia Luo, Peifeng Wei, Feng Miao

**Affiliations:** 1 Department of Traditional Chinese Medicine, Xian XD Group Hospital, Xi’an, Shaanxi 710077, China; 2 Department of Chinese Internal Medicine, Affiliated Hospital of Shaanxi University of Chinese Medicine, Xianyang, Shaanxi 712046, China; 3 College of Pharmacy of Shaanxi University of Chinese Medicine, Xianyang, Shaanxi 712046, China

**Keywords:** AMPK, Emodin, ER-stress, senile dementia, Zinc

## Abstract

Emodin is a natural anthraquinone derivative with numerous beneficial effects, including antioxidant properties, anti-tumor activities, and protecting the nerves. Zinc-induced neurotoxicity plays a crucial role in the pathogenesis of vascular dementia (VD) and Parkinson’s disease (PD). Here, the protective activity of emodin inhibiting zinc-induced neurotoxicity and its molecular mechanisms such as cellular Zn^2+^ influx and zinc-induced gene expression were examined using human neuroblastoma cells (SH-SY5Y cells). Our findings showed that emodin obviously enhanced cell viability and reduced cell apoptosis and lactate dehydrogenase release. Bedsides, we detected a decrease of intracellular Zn^2+^ concentration after SH-SY5Y cells were pretreated with emodin. Simultaneously, the expression of zinc transporter-1, metallothionein-1, and metallothionein-2 were weakened in emodin-pretreated SH-SY5Y cells. In addition, emodin prevented the depletion of NAD+ and ATP induced by zinc. Emodin also reduced intracellular reactive oxygen species and endoplasmic reticulum-stress levels. Strikingly, emodin elevated SH-SY5Y cell viability and inhibited cell apoptosis caused by AMP-activated protein kinase signaling pathway activation. Thus, emodin could protect against neurotoxicity induced by Zn^2+^ in neuroblastoma SH-SY5Y cells. It is expected to have future therapeutic potential for VD or PD and other neurodegenerative diseases.

## Introduction

The incidence of senile dementia is increasing in a rapidly aging world. Approximately 65.7 million people will be living with senile dementia by 2030 [1]. Parkinson’s disease (PD) is a neurodegenerative disease characterized by the progressive loss of dopaminergic neurons in the substantia nigra. Vascular dementia (VD) is a type of dementia due to cerebrovascular lesions. As the pathogenesis underlying VD and PD remains unclear, there are few clinical preventive measures at present. Thus, it is urgently to study the potential pathogenesis of VD or PD and to find drugs to prevent or treat senile dementia.

Studies found that the excessive entry of zinc (Zn^2+^) into neurons is critical to ischemia-induced neuronal death and, eventually, contributes to the pathogenesis of VD and PD [2–4]. Kim and co-workers [5] indicated that elevation of intracellular Zn^2+^ level induced various functional abnormalities of neurons, ultimately leading to the toxicity and death of neurons. Excessive Zn^2+^ has been also reported to reduce cellular nicotinamide adenine dinucleotide (NAD+) level, and thus resulting in inhibition of ATP and failure of energy [6,7]. Furthermore, it has been shown that Zn^2+^ produces reactive oxygen species (ROS) and induces oxidative damage resulting from mitochondrial impairments [8]. Thus, ATP depletion and ROS production in neuron cells characterize zinc-induced neurotoxicity.

Emodin (1, 3, 8-trihydroxy-6-methylan-thraquinone), a natural anthraquinone derivative extracted from polygonum multiflorum, rhubarb, or alose, has been demonstrated to possess multiple biological functions, such as antioxidant [9] and antitumor [10] properties. Increasing evidence have suggested that emodin has neuroprotective effects. Emodin may prevent the formation of atherosclerotic plaques [11,12], reduce neuron cells apoptosis [13], and inhibit glutamate toxicity [14,15]. However, the role and mechanism of emodin in zinc-induced neurotoxicity have not been clarified.

The present study is aimed to investigate the potential protective activity of emodin on zinc-induced neurotoxicity. Furthermore, the potential molecular mechanisms were also explored in SHSY5Y neuroblastoma cells, ultimately providing clues for novel VD and PD treatments.

## Materials and methods

### Cell culture

Human neuroblastoma SH-SY5Y cells (obtained from American Type Culture Collection, Manassas, VA) were grown in complete minimum essential medium supplemented with 10% (v/v) heat-inactivated fetal bovine serum and 100 U/ml streptomycin. After trypsin digestion, cells were resuspended in serum-free medium, distributed into culture dishes, and cultured in a humidified incubator (5% CO_2_) at 37°C.

### Neurotoxicity experiments

Cell viability was assessed as previously described [16]. Dissociated SH-SY5Y cells were distributed into 96-well culture plates at a concentration of 5 × 10^4^ cells per well in 200 µl culture medium. After 24-h incubation, cells were treated with various concentrations of emodin (0, 5, 10, 20, 50 µM) prior to the addition of ZnSO_4_ (200 µM) to the medium for 1 h, the exposing to Zn^2+^ was terminated by replacing Zn^2+^-containing medium with fresh serum-free medium, and then cell viability was quantified using a WST-based cell counting kit (MedChemexpress, Jersey, U.S.A.). Absorbances of treated samples were measured against a blank control using an iMark microplate absorbance reader (Bio-Rad, Hercules, CA, U.S.A.) at 450 nm wavelength. On the other hand, SH-SY5Y cells were fixed with 0.5 ml of 95% (v/v) ethanol, and then rinsed twice with phosphate-buffered saline (PBS). The degree of cellular apoptosis was analyzed via using an ApoBrdU DNA fragmentation assay kit (BioVision, Mountain View, CA, U.S.A) and an annexin V-FITC/PI apoptosis detection kit (Abcam, Cambridge, U.K.), following the manufacturer’s instructions. The apoptotic rate was measured as the percentage of TUNEL-positive cells or FITC+/PI- cells to the total number of cells under 100× magnification.

The lack of membrane integrity associated with necrosis was also assessed by measuring the leakage of lactate dehydrogenase (LDH). The supernatant was collected 1 h after exposing to Zn^2+^, and then cells were lysed in 1% Triton X-100 for the determination of total LDH. The release of LDH was measured by using the LDH-cytotoxicity assay kit (Biovision, San Francisco Bay Area, U.S.A.) as described by the manufacturer.

### Quantification of intracellular Zn^2+^ levels

Concentrations of Zn^2+^ in SH-SY5Y cells were measured using a zinc assay kit (Sigma, St. Louis, MO, U.S.A.), according to the manufacturer’s instruction. SH-SY5Y cells were cultured in 96-well plates as described above. To evaluate the effects of emodin on Zn^2+^ levels, cells were pretreated with 20 µM of emodin for 24 h and exposed to 200 µM of ZnSO_4_ for 1 h.

### RNA extraction and quantitative real-time reverse transcription PCR

Total RNA was extracted from the treated SH-SY5Y cells with TRIzol reagent (Invitrogen, Carlsbad, CA, U.S.A.). First-strand cDNA was synthesized from 4 µg of total RNA. Quantitative real-time reverse transcription PCR (RT-qPCR) was performed to determine expression levels of metallothionein (MT1 and MT2) and ZnT1, as well as endoplasmic reticulum stress-related proteins including CHOP, GADD34, and ATF4. mRNA levels of these proteins were normalized relative to β-actin mRNA level in each sample. RT-qPCR was conducted using SYBR Premix Ex Taq™ (Takara, Dalian, China) at 95°C for 1 min, followed by 35 cycles of 95°C for 20 s and 58°C for 1 min in the ABI StepOnePlus Real-time PCR system. The relative fold changes in mRNA expression were calculated using the 2^−ΔΔCT^ method. The primers used for RT-qPCR analysis were listed in Table 1.

**Table 1:** Primer sequences for RT-qPCR analysis

	Target	Primer sequence (5′→3′)
ZnT-1	Forward	CCCACTGCTCAAGGAGTCCGCTCT
	Reverse	TGTAACTCATGGACTTCCTCCACT
MT1	Forward	GTACCTTCTCCTCACTTACTCCG
	Reverse	GTATAGGAAGACGCTGGGTTGG
MT2	Forward	ATGGACCCCAACTGCTCCTG
	Reverse	CAGCAGGTGCACTTGTCCGA
CHOP	Forward	CCACCACACCTGAAAGCAGAA
	Reverse	AGGTGA AAGGCAGGGACTCA
GADD34	Forward	CCTCTA AAAGCTCGGAAGGTACAC
	Reverse	TCGGACTGTGGA AGAGATGGG
ATF4	Forward	GGGTTCTGTCTTCCACTCCA
	Reverse	AAGCAGCAGAGTCAGGCTTTC
β-Actin	Forward	CGCATCCTCTTCCTCCCTGG
	Reverse	CCTAGAAGCACT TGCGGTGCAC

### Protein extraction and Western blot analysis

SH-SY5Y cells grown in 12-well culture plates (1 × 10^6^ cells/well) were lysed with 200 µl 10 mM TRIzol (Invitrogen, CA, U.S.A.). Total proteins from each lysate were separated by SDS-PAGE and transferred onto PVDF membranes and then blocked with 5% non-fat milk for 1 h. Following by probing with the indicated primary antibodies at 4°C with gentle shaking overnight and horseradish peroxidase (HRP)-conjugated secondary antibodies (Abcam, Cambridge, MA, U.S.A.). The proteins were visualized by chemiluminescence, and signals were quantified by ImageJ software. Antibodies used in the present study are as follows p-ACC (CST, Boston, MA, U.S.A.; cat. no. 3661); ACC (CST, Boston, MA, U.S.A.; cat. no. 3662); p-AMPKα (CST, Boston, MA, U.S.A.; cat. no. 2535); AMPKα (CST, Boston, MA, U.S.A.; cat. no. 2603). The dilution factor for antibodies was 1:500.

### Measurement of NAD+

Intracellular NAD+ levels were measured using the EnzyChrom NAD+/NADH assay kit (BioAssay Systems, Hayward, CA, U.S.A.). SH-SY5Y cells were washed with PBS, and then lysed with the supplied NAD extraction buffer. NAD+ was extracted from the lysate according to the manufacturer’s protocol. The measurement of NAD+ is based on an alcohol dehydrogenase cycling reaction. The change in absorbance at 565 nm for 15 min at room temperature was measured.

### Measurement of ATP

SH-SY5Y cells were treated with emodin (20 µM) for 24 h, and then the culture medium was removed, stored, and replaced by HEPES buffer. Cells were incubated with ZnSO_4_ (200 µM) for 1 h at 37°C in HEPES buffer. After washing, the initially stored culture medium was added to cells for 1 h. The cells were lysed with 10 mM Tris-HCl (pH 7.8), and ATP content was determined using a quantitative bioluminescent assay (Sigma, St. Louis, MO, U.S.A.) according to the instructions of the manufacturer and an iMark microplate absorbance reader (Bio-Rad, Hercules, CA, U.S.A.).

### ROS detection

SH-SY5Y cells were digested (5 × 10^6^/ml) and incubated in 10 µM DCFH-DA (Sigma, St. Louis, MO, U.S.A.) probe diluted by the serum-free medium at 37°C for 20 min. After being washed for three times, the cells were tested on iMark microplate absorbance reader (Bio-Rad, Hercules, CA, U.S.A.) using 488 nm excitation wavelength.

### Statistical analysis

The data are expressed as means ± SEM and each experiment was performed in triplicate in the present study. After the homogeneity test for variance, comparisons between groups were performed by one-way analysis of variance (ANOVA) using SPSS 22.0 software, and then post-hoc test was determined by LSD test. A significant difference was indicated when the *P*-value < 0.05.

## Result

### Protective activity of emodin against zinc-induced neurotoxicity

**Figure 1:**
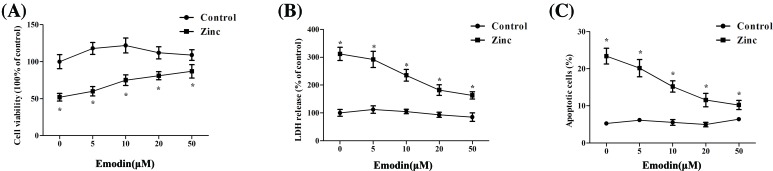
Protective effects of emodin against zinc-induced neurotoxicity Neuroblastoma SH-SY5Y cells were pretreated with various concentrations of emodin (0.5, 10, 20, 50 μM) for 24 h, and then were exposed to ZnSO_4_ (200 μM) for 1 h in serum-free medium. (**A**) The cell viability rate was determined using the water-soluble tetrazolium salt (WST-8): viability rate (%) = (absorbance at 450 nm in the experimental group / absorbance at 450 nm in the control group) × 100%. (**B**) LDH release was measured in the same experimental conditions and expressed as a percent of total LDH activity after complete cell lysis. (**C**) The percentage of apoptotic cells was determined using an annexin V-FITC/PI apoptosis detection kit. The polylines were presented as the mean ± SEM (*n* ≥ 3). *P<0.05 vs. SH-SY5Y cells treated with emodin alone.

To investigate whether emodin exerts protective effects against zinc-induced neurotoxicity, neuroblastoma SH-SY5Y cells were pretreated with various concentrations of emodin (0.5, 10, 20, 50 μM) for 24 h, followed by exposing to ZnSO_4_ (200 μM) for 1 h. Our results showed that emodin obviously reduced SH-SY5Y cell apoptosis under different concentrations. As shown in Figure 1A, pretreatment with emodin rescued SH-SY5Y cells from Zn^2+^ neurotoxicity. Cell viability of SH-SY5Y cells was improved with increasing concentration of emodin. Additionally, LDH release and the level of SH-SY5Y cell apoptosis were decreased due to the pretreatment of emodin (Figure 1B,C). Furthermore, the protective activity of emodin against zinc-induced neurotoxicity reached a plateau at a concentration of 20 μM (Figure 1A–C). Based on these observations, 20 μM of emodin was used in the following experiments.

### Emodin suppressed Zn^2+^ influx into SH-SY5Y cells

**Figure 2:**
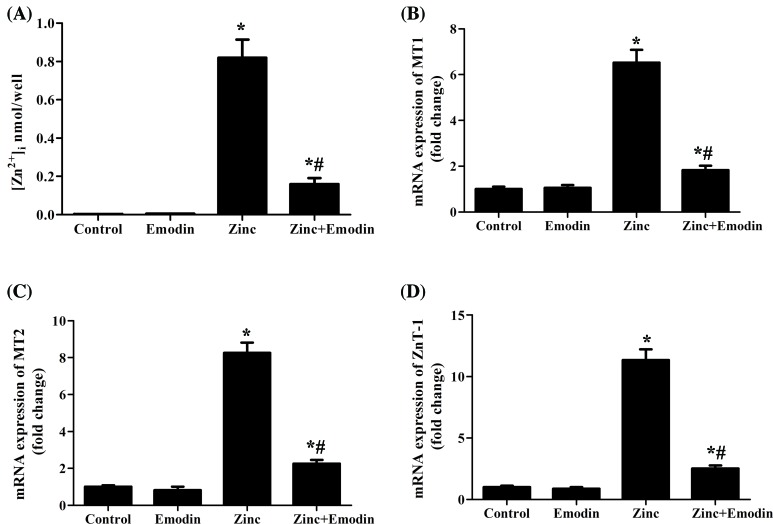
Emodin (20 μM) suppressed Zn^2+^ influx into SH-SY5Y cells SH-SY5Y cells were pretreated with or without emodin (20 µM) for 24 h, and then the cells were exposed to ZnSO_4_ (200 µM) for 1 h in serum-free medium. Concentration of Zn^2+^ (**A**) was measured as described in ‘Materials and methods’ section. The expression of zinc transporter families MT1 (**B**), MT2 (**C**), and ZnT-1 (**D**) was analyzed by RT-qPCR. The columns were presented as the mean ± SEM (*n* ≥ 3). *P<0.05 vs. untreated SH-SY5Y cells or SH-SY5Y cells treated with emodin alone. ^#^*P*<0.05 vs. SH-SY5Y cells treated with zinc alone.

Entry of Zn^2+^ into neurons is thought to be a critical step of Zn^2+^ neurotoxicity [17,18]. Therefore, we examined whether emodin affected the entry of Zn^2+^ into SH-SY5Y cells. The results implied that emodin decreased the elevation of intracellular Zn^2+^ levels induce by Zn^2+^ exposure (Figure 2A). Furthermore, we analyzed the expression of zinc transporter families including MT1, MT2 and metal-binding proteins such as metallothionein 1(MT1) genes in SH-SY5Y cells. Our findings showed that the mRNA levels of MT1 (Figure 2B), MT2 (Figure 2C), and ZnT-1 (Figure 2D) were significantly increased after 1 h of Zn^2+^ treatment, while emodin markedly attenuated this induction.

### Emodin prevents zinc-induced depletion of intracellular NAD+ and ATP

**Figure 3:**
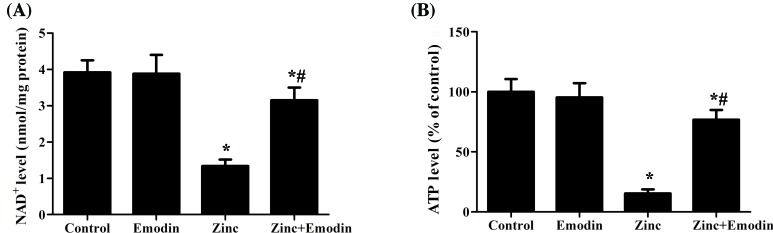
Effects of emodin on Zn^2+^-induced NAD+ depletion and ATP depletion SH-SY5Y cells were pretreated with or without emodin (20 µM) for 24 h, and then SH-SY5Y cells were exposed to ZnSO_4_ (200 µM) for 1 h in serum-free medium. After Zn^2+^ exposure, intracellular NAD+ (**A**) and ATP (**B**) contents were measured. Neuronal ATP content was expressed as a percent of the amount of ATP measured in SH-SY5Y cells exposed to sham treatment. The columns were presented as the mean ± SEM (*n*≥3). *P<0.05 vs. untreated SH-SY5Y cells or SH-SY5Y cells treated with emodin alone. ^#^*P*<0.05 vs. SH-SY5Y cells treated with zinc alone.

It has been widely recognized that Zn^2+^ overload in neurons can cause reduction of intracellular NAD+ and ATP [6,7]. Therefore, to examine the effects of emodin on zinc-induced NAD+ and ATP depletion, we measured intracellular NAD+ and ATP levels. As shown in Figure 3A,B, Zn^2+^ drastically suppressed intracellular NAD+ and ATP levels. Pretreatment with emodin significantly prevented the depletion of NAD+ (Figure 3A) and ATP (Figure 3B) caused by 200 µM Zn^2+^ exposure.

### Emodin against zinc-induced production of reactive oxygen species and endoplasmic reticulum-stress

**Figure 4:**
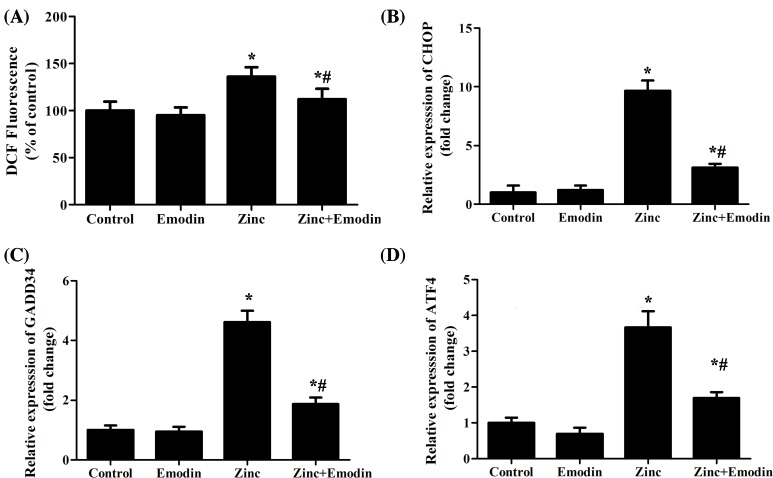
Emodin against zinc-induced production of reactive oxygen species (ROS) and endoplasmic reticulum-stress SH-SY5Y cells were treated with 200 µM ZnSO_4_ for 1 h in the absence or presence of emodin (20 µM). (**A**) Detection of ROS content using fluorescent probe DCFH-DA. Expression of CHOP (**B**), GADD34 (**C**), and ATF4 (**D**) were analyzed by RT-qPCR. The columns were presented as the mean ± SEM (*n*≥3). *P<0.05 vs. untreated SH-SY5Y cells or SH-SY5Y cells treated with emodin alone. ^#^*P*<0.05 vs. SH-SY5Y cells treated with zinc alone.

We then detected the ROS content, which is related to oxidative stress, neurodegeneration and aging [19]. The data showed that treatment with ZnSO_4_ promoted generation of ROS in SH-SY5Y cells, and emodin obviously suppressed zinc-induced ROS production (Figure 4A). On the other hand, it is widely accepted that ER-stress causes cell death via the accumulation of misfolded or unfolded proteins [20]. ER-stress is related to the pathogenesis of various neurodegenerative diseases such as PD, Alzheimer’s disease (AD), and ischemia-induced neurodegeneration [21,22]. Therefore, we also examined the mRNA levels of three proteins related to ER-stress, including C/EBP homologous protein (CHOP), growth arrest and DNA damage inducible gene 34 (GADD34), and activating transcription factor 4 (AFT4). Our findings displayed that the expression of CHOP (Figure 4B), GADD34 (Figure 4C), and ATF4 (Figure 4D) were obviously enhanced compared with the control groups after exposed to Zn^2+^. Fortunately, emodin significantly decreased the zinc-induced CHOP, GADD34, and ATF4 protein expression.

### Zinc-induced AMPK/ACC activation was inhibited by emodin

**Figure 5:**
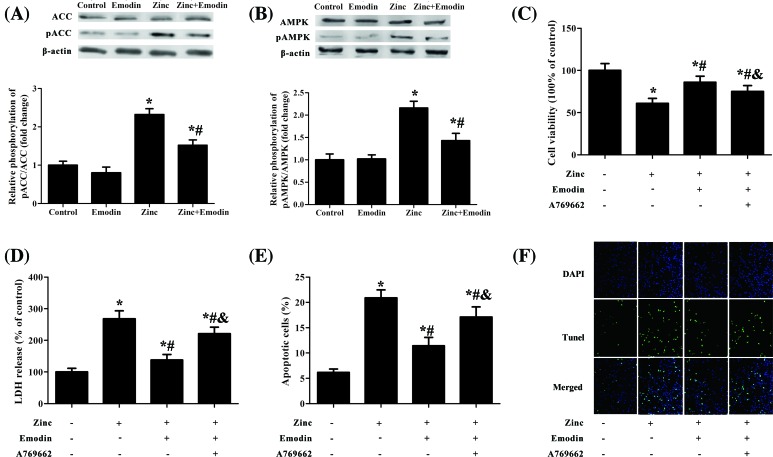
Zinc-induced AMPK activation was inhibited by emodin SH-SY5Y cells were treated with 200 µM of ZnSO_4_ for 1 h in the absence or presence of emodin (20 µM), or/and A769662. A769662 is an activator of the AMPK signaling pathway. Western blot analysis results to document phosphorylation of ACC (**A**) and AMPK (**B**) after zinc treatment. (**C**) The cell viability rate was determined using the water-soluble tetrazolium salt (WST-8). (**D**) LDH release was measured in the same experimental conditions and expressed as a percent of total LDH activity after complete cell lysis. (**E**) The percentage of apoptotic cells was determined using an annexin V-FITC/PI apoptosis detection kit. (**F**) The percentage of apoptotic cells was determined using an ApoBrdU DNA fragmentation assay kit. The columns were presented as the mean ± SEM (*n*≥3). *P<0.05 vs. untreated SH-SY5Y cells or SH-SY5Y cells treated with emodin alone. ^#^*P*<0.05 vs. SH-SY5Y cells treated with zinc alone. ^&^*P*<0.05 vs. SH-SY5Y cells treated with zinc and emodin.

Studies have shown that AMPK and ACC were involved in cytotoxicity and cell death [23–25]. Thus, we further investigated the AMPK and ACC expression of zinc-exposed SH-SY5Y cells in the presence or absence of emodin (20 μM). The Western blot analysis displayed that the phosphorylation levels of ACC (Figure 5A) and AMPK (Figure 5B) in SH-SY5Y cells pretreated with emodin were obviously lower than that treated with Zn^2+^ alone. In addition, activation of the AMPK signaling pathway using AMPK signaling pathway activator (A769662) partially weakened the protective activity of emodin against zinc-induced neurotoxicity (Figure 5C–F). These findings suggest that emodin partially avoids zinc-induced neuronal death through regulating the AMPK signaling pathway.

## Discussion

Emodin has a significant role in preventing hypoxic-ischemic neuronal injury [26]. Gu et al. [15] suggested that emodin inhibited the excitatory postsynaptic potential by decreasing the release of glutamate. Moreover, Ca^2+^ signal transduction of smooth muscle cells and Ca^2+^ concentration of cardiomyocytes could be regulated by emodin [27,28]. Notably, recent researches showed that Ca^2+^ and Zn^2+^ synergistically caused neurotoxicity [29]. However, the role and mechanism of emodin in zinc-induced neurotoxicity have not been elucidated. This investigation shows that emodin reduces apoptosis induced by excessive Zn^2+^ in human neuroblastoma SH-SY5Y cells with elevation of cellular ATP and suppression of ROS and ER-stress and the AMPK signaling pathway (Figures 1–5).

To date, entry of Zn^2+^ into neurons has been thought to be a critical step of Zn^2+^ neurotoxicity [17]. Zn^2+^ transport into or out of neurons has been reported to be regulated by various molecules including calcium‐permeable AMPA/Kainate channels [30], transient receptor potential channels (TRP channels) [31], Na^+^/Zn^2+^ exchanger [32], and zinc transporters [18]. Evidence suggests that zinc transporter families, ZIP, ZnTs, and metal-binding proteins such as metallothionein (MT) play important roles in Zn^2+^ homeostasis in the brain [18]. ZnT-1 [33], MT1, and MT2 [34] were used to transport and eliminate excess zinc in the cytoplasm, and may be associated with toxic Zn^2+^ levels. We demonstrated here that emodin decreased the concentration of intracellular Zn^2+^ after Zn^2+^ exposure and weakened the expression levels of MT1, MT2, and ZnT-1 mRNAs in cultured SH-SY5Y cells. Thus, the suppressed expression of ZnT-1, MT1, and/or MT2 in SH-SY5Y cells could partly explain emodin suppressed Zn^2+^ influx into neuron cells.

Mounting evidence have pointed out that excessive accumulation of Zn^2+^ in neurons causes reduction of cellular NAD+ and inhibition of GAPDH [6,7]. Intracellular NAD+ level is known to be a critical determinant of neuronal survival [35]. It was reported that addition of exogenous NAD+ reduced zinc-induced neurotoxicity [35]. Additionally, Zn^2+^ induced a progressive decline of ATP level and energy failure in the cytoplasm [36]. Our finding revealed that pretreatment with emodin ameliorated a decrease in intracellular NAD+ and ATP levels caused by Zn^2+^ exposure. These results suggest that emodin-induced reduction in intracellular Zn^2+^ accumulation protects against zinc-induced neurotoxicity via rescuing the generation of NAD+ and ATP.

On the other hand, it has been suggested that intracellular Zn^2+^ could mediate ER-stress [37]. ER-stress is caused by the accumulation of unfolded or misfolded proteins in the ER and increase in production of ROS [38]. Evidence revealed that Zn^2+^ entry into cytoplasm through the mitochondrial Ca^2+^ uniporter resulted in mitochondrial dysfunction and ROS generation [39]. Moreover, excess accumulation of ROS and destruction of Zn^2+^ homeostasis might lead to neurodegenerative diseases [40]. Our findings supported that emodin significantly inhibited ROS generation and decreased the zinc-induced CHOP, GADD34, and ATF4 protein overexpression, implying that emodin may protect against zinc-induced neurotoxicity in neuroblastoma SH-SY5Y cells by blocking the production of ROS and ER-stress.

Several studies demonstrate that AMPK is a regulator of cellular energy homeostasis [41] and is related to neuronal survival and death especially under ATP deprivation [42]. Excitotoxicity or ischemic brain injury in a rat model was significantly attenuated by the inhibition of AMPK and ACC activity [25,43,44], which suggested a role of AMPK and ACC in neurotoxicity. Our present study showed that AMPK/ACC were activated in zinc-induced neurotoxicity in neuroblastoma SH-SY5Y cells. Furthermore, AMPK activation caused a negative impact on emodin-pretreated neuron cell viability, which indicated that emodin improved zinc-induced neurons apoptosis and increased neurons viability via inhibiting the AMPK signaling pathway.

In summary, we provided evidence that emodin could inhibit the influx of Zn^2+^ into neuronal cells, and the decrease of intracellular Zn^2+^ level prevented the depletion of NAD+ and ATP, inhibited the generation of ROS and ER-stress, and inactivated the AMPK/ACC signaling pathway, thus inhibiting neuroblastoma SH-SY5Y cell apoptosis and playing a neuroprotective role. Accordingly, emodin may be useful as a drug for the treatment of VD or PD.

## Abbreviations

AFT4: activating transcription factor 4
CHOP: C/EBP homologous protein
GADD34: growth arrest and DNA damage inducible gene 34
LDH: lactate dehydrogenase
PD: Parkinson’s disease
ROS: reactive oxygen species
VD: vascular dementia

## References

[1] Prince M. World Alzheimer report 2015: the global impact of dementia. https://www.alz.co.uk/research/word-report-2015

[2] Weiss J.H., Sensi S.L. and Koh J.Y. (2000) Zn2+: a novel ionic mediator of neural injury in brain disease. Trends Pharmacol. Sci. 21, 395–401 10.1016/S0165-6147(00)01541-8 11050320

[3] Lobner D., Canzoniero L.M., Manzerra P., Gottron F., Ying H., Knudson M. (2000) Zinc-induced neuronal death in cortical neurons. Cell. Mol. Biol. (Noisy-le-grand) 46, 797–806 10875441

[4] Tian K., He C.C., Xu H.N., Wang Y.X., Wang H.G., An D. (2017) Zn 2+ reduction induces neuronal death with changes in voltage-gated potassium and sodium channel currents. J. Trace Elem. Med. Biol. 41, 66–74 10.1016/j.jtemb.2017.02.011 28347465

[5] Luo L., Kim S.W., Lee H.K., Kim I.D., Lee H. and Lee J.K. (2017) Anti-Zn 2+ -toxicity of 4-hydroxybenzyl alcohol in astrocytes and neurons contribute to a robust neuroprotective effects in the postischemic brain. Cell. Mol. Neurobiol. 1–12 28608001 10.1007/s10571-017-0508-y PMC11481900

[6] Sheline C.T., Behrens M.M. and Choi D.W. (2000) Zinc-induced cortical neuronal death: contribution of energy failure attributable to loss of NAD(+) and inhibition of glycolysis. J. Neurosci. 20, 3139–3146 10.1523/JNEUROSCI.20-09-03139.2000 10777777 PMC6773145

[7] Cai A.L., Zipfel G.J. and Sheline C.T. (2006) Zinc neurotoxicity is dependent on intracellular NAD levels and the sirtuin pathway. Eur J Neurosci. Eur. J. Neurosci. 24, 2169–2176 10.1111/j.1460-9568.2006.05110.x 17042794

[8] Sensi S.L., Ton-That D., Sullivan P.G., Jonas E.A., Gee K.R., Kaczmarek L.K. (2003) Modulation of mitochondrial function by endogenous Zn pools. Proc. Natl. Acad. Sci. U.S.A. 100, 6157–6162 10.1073/pnas.1031598100 12724524 PMC156342

[9] Jaesue C. (2000) Comparative evaluation of antioxidant potential of alaternin (2-hydroxyemodin) and emodin. J. Agric. Food Chem. 48, 6347–6351 10.1021/jf000936r 11312806

[10] Braumann C., Koplin G., Geier C., Höhn P., Pohlenz J., Dubiel W. (2017) Dose-dependent role of novel agents emodin and BTB14431 in colonic cancer treatment in rats. Acta Chir. Belg. 117, 1 28669313 10.1080/00015458.2017.1341145

[11] Heo S.K., Yun H.J., Noh E.K. and Park S.D. (2010) Emodin and rhein inhibit LIGHT-induced monocytes migration by blocking of ROS production. Vasc. Pharmacol. 53, 28–37 10.1016/j.vph.2010.03.002 20298810

[12] Li J.S., Liu J.X., Liang S.W., Zhao J.M., Liu K. and Wang M.H. (2004) Effects of glucoside and aglycone parts of rhubarb on the metabolism of free radicals in rats with ischemic brain injury. Chin. J. Clin. Rehab. 8, 7748–7750

[13] Huang Z., Chen G. and Shi P. (2008) Emodin-induced apoptosis in human breast cancer BCap-37 cells through the mitochondrial signaling pathway. Arch. Pharm. Res. 31, 742–748 10.1007/s12272-001-1221-6 18563356

[14] Wang C., Zhang D., Ma H. and Liu J. (2007) Neuroprotective effects of emodin-8- O -β- d -glucoside in vivo and in vitro. Eur. J. Pharmacol. 577, 58–63 10.1016/j.ejphar.2007.08.033 17897641

[15] Gu J.W., Hasuo H., Takeya M. and Akasu T. (2005) Effects of emodin on synaptic transmission in rat hippocampal CA1 pyramidal neurons in vitro. Neuropharmacology 49, 103–111 10.1016/j.neuropharm.2005.02.003 15992585

[16] Kawahara M., Sadakane Y., Koyama H., Konoha K. and Ohkawara S. (2013) D-histidine and L-histidine attenuate zinc-induced neuronal death in GT1-7 cells. Metallomics 5, 453–460 10.1039/c3mt20264j 23503404

[17] Mizuno D. and Kawahara M. (2013) The molecular mechanisms of zinc neurotoxicity and the pathogenesis of vascular type senile dementia. Int. J. Mol. Sci. 14, 22067–22081 10.3390/ijms141122067 24213606 PMC3856052

[18] Colvin R.A., Fontaine C.P., Laskowski M. and Thomas D. (2003) Zn2+ transporters and Zn2+ homeostasis in neurons. Eur. J. Pharmacol. 479, 171–185 10.1016/j.ejphar.2003.08.067 14612148

[19] Sesti F., Liu S. and Cai S.Q. (2010) Oxidation of potassium channels by ROS: a general mechanism of aging and neurodegeneration? Trends Cell Biol. 20, 45–51 10.1016/j.tcb.2009.09.008 19850480

[20] Brown M.K. and Nirinjini N. (2012) The endoplasmic reticulum stress response in aging and age-related diseases. Front. Physiol. 3, 263 10.3389/fphys.2012.00263 22934019 PMC3429039

[21] Ferreiro E., Baldeiras I., Ferreira I.L., Costa R.O., Rego A.C., Pereira C.F. (2012) Mitochondrial- and endoplasmic reticulum-associated oxidative stress in Alzheimer’s disease: from pathogenesis to biomarkers. Int. J. Cell Biol. 2012, 735206 10.1155/2012/735206 22701485 PMC3373122

[22] Roussel B.D., Kruppa A.J., Miranda E., Crowther D.C., Lomas D.A. and Marciniak S.J. (2013) Endoplasmic reticulum dysfunction in neurological disease. Lancet Neurol. 12, 105–118 10.1016/S1474-4422(12)70238-7 23237905

[23] Santosh R. and Gabriele R. (2012) AMP-Activated Protein Kinase (AMPK) and Energy-Sensing in the Brain. Exp. Neurobiol. 21, 52–60 10.5607/en.2012.21.2.52 22792025 PMC3381212

[24] Nakatsu Y., Kotake Y., Hino A. and Ohta S. (2008) Activation of AMP-activated protein kinase by tributyltin induces neuronal cell death. Toxicol. Appl. Pharmacol. 230, 358–363 18511093 10.1016/j.taap.2008.03.021

[25] Concannon C.G., Tuffy L.P., Weisová P., Bonner H.P., Dávila D., Bonner C. (2010) AMP kinase—mediated activation of the BH3-only protein Bim couples energy depletion to stress-induced apoptosis. J. Cell Biol. 189, 83 10.1083/jcb.200909166 20351066 PMC2854380

[26] Guo H., Shen X., Xu Y., Yuan J., Zhao D. and Hu W. (2013) Emodin prevents hypoxic-ischemic neuronal injury Involvement of the activin A pathway. Neural Regen. Res. 8, 1360–1367 25206430 10.3969/j.issn.1673-5374.2013.15.002 PMC4107762

[27] Ma T., Qi Q.H., Yang W.X., Xu J. and Dong Z.L. (2003) Contractile effects and intracellular Ca∼(2+)signalling induced by emodin in circular smooth muscle cells of rat colon. World J. Gastroenterol. 9, 1804 10.3748/wjg.v9.i8.1804 12918125 PMC4611548

[28] Liu Y., Shan H.L., Sun H.L., Shu zhuang H.E. and Yang B. (2004) Effects of emodin on the intracellular calcium concentration ([Ca∼(2+)]_i) and L-type calcium current of the single ventricular mytocytes from guinea pig. Acta Pharm. Sin. 39, 5–8 15127572

[29] Tanaka K.I. and Kawahara M. (2017) Copper enhances zinc-induced neurotoxicity and the endoplasmic reticulum stress response in a neuronal model of vascular dementia. Front. Neurosci. 11, 58 10.3389/fnins.2017.00058 28232787 PMC5299027

[30] Qin Y. (2008) Studies of zinc transport and its contribution to zinc homeostasis in cultured cortical neurons. Dissert. Theses - Gradworks

[31] Inoue K., Branigan D. and Xiong Z.G. (2010) Zinc-induced Neurotoxicity Mediated by Transient Receptor Potential Melastatin 7 Channels. J. Biol. Chem. 285, 7430 10.1074/jbc.M109.040485 20048154 PMC2844191

[32] Ohana E., Segal D., Palty R., Tonthat D., Moran A., Sensi S.L. (2004) A sodium zinc exchange mechanism is mediating extrusion of zinc in mammalian cells. J. Biol. Chem. 279, 4278–4284 10.1074/jbc.M309229200 14581475

[33] Mcmahon R.J. and Cousins R.J. (1998) Regulation of the zinc transporter ZnT-1 by dietary zinc. Proc. Natl. Acad. Sci. U.S.A. 95, 4841–4846 10.1073/pnas.95.9.4841 9560190 PMC20175

[34] Maret W., Hambidge M., Cousins R.J. and Costello R.B. (2000) The function of zinc metallothionein: a link between cellular zinc and redox state. J. Nutr. 130, 1455S 10.1093/jn/130.5.1455S 10801959

[35] Cai A.L., Zipfel G.J. and Sheline C.T. (2006) Zinc neurotoxicity is dependent on intracellular NAD levels and the sirtuin pathway. Eur. J. Neurosci. 24, 2169 10.1111/j.1460-9568.2006.05110.x 17042794

[36] Dineley K.E., Votyakova T.V. and Reynolds I.J. (2003) Zinc inhibition of cellular energy production: implications for mitochondria and neurodegeneration. J. Neurochem. 85, 563–570 10.1046/j.1471-4159.2003.01678.x 12694382

[37] Tuncay E., Bitirim V.C., Durak A., Grj C., Taylor K.M., Rutter G.A. (2017) Hyperglycemia-Induced Changes in ZIP7 and ZnT7 Expression Cause Zn(2+) Release From the Sarco(endo)plasmic Reticulum and Mediate ER Stress in the Heart. Diabetes 66, 1346 10.2337/db16-1099 28232492

[38] Zeeshan H.M., Lee G.H., Kim H.R. and Chae H.J. (2016) Endoplasmic Reticulum Stress and Associated ROS. Int. J. Mol. Sci. 17, 327 10.3390/ijms17030327 26950115 PMC4813189

[39] Medvedeva Y.V. and Weiss J.H. (2014) Intramitochondrial Zn2+ accumulation via the Ca2+ uniporter contributes to acute ischemic neurodegeneration. Neurobiol. Dis. 68, 137–144 10.1016/j.nbd.2014.04.011 24787898 PMC4065779

[40] Fernández-Checa J.C., Fernández A., Morales A., Marí M., García-Ruiz C. and Colell A. (2010) Oxidative stress and altered mitochondrial function in neurodegenerative diseases: lessons from mouse models. CNS Neurol. Disorders Drug Targets 9, 439–54 10.2174/187152710791556113 20522012

[41] Huynh M.K., Kinyua A.W., Yang D.J. and Kim K.W. (2016) Hypothalamic AMPK as a regulator of energy homeostasis. Neural Plast. 2016, 2754078 10.1155/2016/2754078 27547453 PMC4980534

[42] Qiu F., Zhang H., Yuan Y., Liu Z., Huang B., Miao H. (2018) A decrease of ATP production steered by PEDF in cardiomyocytes with oxygen-glucose deprivation is associated with an AMPK-dependent degradation pathway. Int. J. Cardiol. 257, 10.1016/j.ijcard.2018.01.034 29361350

[43] Li J. and Mccullough L.D. (2010) Effects of AMP-activated protein kinase in cerebral ischemia. J. Cereb. Blood Flow Metab. 30, 480–492 10.1038/jcbfm.2009.255 20010958 PMC2852687

[44] Li M., Zhao J., Hu Y., Lu H. and Guo J. (2010) Oxygen free radicals regulate energy metabolism via AMPK pathway following cerebral ischemia. Neurol. Res. 32, 779–784 10.1179/174313209X459174 19660198

